# 超高效液相色谱-四极杆/静电场轨道阱高分辨质谱法测定毒蘑菇中5种鹅膏肽类毒素

**DOI:** 10.3724/SP.J.1123.2022.03010

**Published:** 2023-01-08

**Authors:** Liying HE, Xiaoqin TANG, Jian ZHAO, Qianzhan YANG, Li LI

**Affiliations:** 1.重庆市疾病预防控制中心, 重庆 400042; 1. Chongqing Center for Disease Control and Prevention, Chongqing 400042, China; 2.岛津企业管理(中国)有限公司重庆分公司, 重庆 400010; 2. Shimadzu (China) Co., Ltd. Chongqing Branch, Chongqing 400010, China

**Keywords:** 超高效液相色谱-四极杆/静电场轨道阱高分辨质谱, 鹅膏毒肽, 鬼笔毒肽, 毒蘑菇, ultra performance liquid chromatography-quadrupole electrostatic field orbitrap high resolution mass spectrometry (UPLC-Q/Orbitrap HRMS), amatoxins, phalloidin, poisonous mushroom

## Abstract

鹅膏肽类毒素是毒蘑菇中毒事件中最常见的致死毒素,因此研究建立了超高效液相色谱-四极杆/静电场轨道阱高分辨质谱检测毒蘑菇中5种鹅膏肽类毒素的方法。样品经纯水提取后,以乙腈-5 mmol/L乙酸铵水溶液(含0.1%甲酸)为流动相进行梯度洗脱,用HSS T3色谱柱(100 mm×2.0 mm, 2.1 μm)对待测组分进行色谱分离;采用可加热电喷雾电离源(HESI),全扫描/数据依赖二级质谱扫描(Full mass-ddMS^2^)模式对待测物进行定性分析;在目标离子选择性扫描(Targeted-SIM)模式下,以外标法对待测物进行定量测定。结果显示,5种鹅膏肽类毒素在1.0~20.0 μg/L范围内均呈现良好的线性关系,相关系数均大于0.99,检出限均为0.006 mg/kg,加标回收率为81.8%~102.4%,相对标准偏差为3.2%~8.3%。方法提供了丰富的化合物特征信息,可根据提取离子流色谱图结合同位素分布信息锁定可疑化合物,根据一级质谱和二级质谱碎片离子的精确质荷比,在没有相关标准品的情况下可对未知化合物进行结构推断和确证。方法样品前处理简单,定性分析特异性强,定量测定灵敏度高,可满足突发公共卫生事件快速定性定量的检测要求,同时也为开展此类毒素中毒快速筛查及未知毒素的结构锁定提供了可靠的技术支撑。

## Abstract (en)

Food poisoning by toxic mushrooms occurs frequently worldwide. It is one of the most common food poisoning events and the main cause of death. Amanita peptide toxins are the most common lethal toxins in poisonous mushrooms. Presently, a novel method based on ultra performance liquid chromatography-quadrupole electrostatic field orbitrap high resolution mass spectrometry (UPLC-Q/Orbitrap HRMS) was developed for the determination of five amanitapeptide toxins (*α*-amanitin, *β*-amanitin, *γ*-amanitin, phalloidin, and phallacidin). Because the isotope summit of *α*-amanitin affects the detection of *β*-amanitin, it cannot be distinguished by low resolution mass spectrometry. Therefore, experimental conditions including chromatography and mass spectrometry were explored in detail. The five peptide toxins were extracted from poisonous mushrooms with pure water and filtered through a 0.22 μm teflon microporous membrane. The procedure was rapid, simple, and environmentally friendly. Chromatographic separation was performed on a strong polarity HSS T3 column (100 mm×2.0 mm, 2.1 μm) with gradient elution using acetonitrile and 5 mmol/L ammonium acetate containing 0.1% (v/v) formic acid as mobile phases at a flow rate of 0.3 mL/min. The column temperature was set to 40 ℃. The analytes were ionized using a heating electrospray ionization source and collected in positive ion mode. Full scanning/data-dependent secondary mass spectrometry (Full mass-ddMS^2^) mode was used for qualitative analysis of the targets within 10 min. The target ion selective scan (Targeted-SIM) mode was used for quantification by external standard calibration. The measured and theoretical values of the exact mass and the MS^2^ fragment ions of the five compounds were within an error of 5×10^-6^. Method validation was performed according to the criteria recommended by the Chinese National Standard. All the compounds showed an excellent linear relationship in the range of 1.0-20.0 μg/L. The correlation coefficients (*r*) ranged from 0.9974 to 0.9989. The limit of detection was 0.006 mg/kg for all five compounds. Recoveries ranged from 81.8% to 102.4%. There was no matrix effect in the blank mushroom sample for the five compounds, and the relative standard deviations ranged from 3.2% to 8.3%. This method provides abundant compound characteristic mass information, such as retention time, exact mass, fragment ions, and other information. The data can be used to identify suspected compounds based on the extracted ion flow diagram and isotope distribution information. Comparison between the actual exact mass and the theoretical exact mass, combined with the fragment ions enables identification of the structures of unknown compounds and collision methods, which can be confirmed in the absence of standard materials. In this study, the isomer of *γ*-amanitin was identified as amaninamide. The novel method is simple, accurate, specific, and sensitive. The method permits the rapid qualitative and quantitative detection of compound in public health emergency settings and will provide reliable technical support for the rapid screening of such toxic compounds and the structural locking of unknown toxins in the future.

毒蘑菇亦称毒蕈、毒菌等,人或畜禽食用后能产生中毒反应^[1]^。我国已知的毒蘑菇有435种^[2]^,每年因误采食毒蘑菇而中毒的事件屡见不鲜,其导致死亡人数占整个食物中毒死亡人数的比例超过35%^[3]^,高达70.49%的毒蘑菇中毒事件由误食剧毒鹅膏菌引起^[4]^。毒蘑菇的毒素可分为环肽类、奥来毒素、毒蕈碱类等,引起中毒的主要是鹅膏肽类毒素,其属于环肽类毒素,为最主要致死毒素,常存在于鹅膏属、环柄菇属的部分品种中^[5]^。鹅膏肽类毒素化学性质稳定,耐高温,一般的烹调加工不会破坏其毒性,由该类毒素引起的中毒死亡病例占毒蘑菇中毒死亡病例的90%以上^[6]^,且目前尚未发现特效解毒剂,因此掌握鹅膏肽类毒素的快速检测技术和方法显得尤为重要。根据鹅膏肽类毒素的氨基酸组成和结构,分为鹅膏毒肽、鬼笔毒肽和毒伞素3类^[7]^。毒伞素致命性相对较弱,相关研究较少。鹅膏毒肽为双环八肽,以*α*-鹅膏毒肽(*α*-AMA)、*β*-鹅膏毒肽(*β*-AMA)和*γ*-鹅膏毒肽(*γ*-AMA)3种毒素毒性最大,在毒蘑菇中含量也较高,为引起中毒的主要毒素,人的致死剂量约为0.1 mg/kg^[8]^。鬼笔毒肽为双环七肽,主要有二羟鬼笔毒肽(POD)、羧基二羟鬼笔毒肽(PCD),鬼笔毒肽类毒素不能经消化道吸收,但含有鬼笔毒肽类毒素的蘑菇多同时含有鹅膏毒肽,因此一般也同时作为监测目标^[9⇓⇓-12]^。

鹅膏毒肽早期的检测方法主要有化学显色法^[13,14]^、酶联免疫吸附法^[15,16]^、毛细管电泳法^[17,18]^、高效液相色谱法^[19,20]^等。近年来液相色谱-串联质谱法^[21⇓⇓-24]^逐渐成为主流检测方法,该法取样量少,较前述其他方法灵敏度更高,定性定量能力更好,但该法的质量分析器以低分辨率和整数质量精度的四极杆为主,毒蘑菇样品通常含有较多的多糖、氨基酸、多肽及有机酸等复杂基质,需要较为繁琐的前处理操作以排除基质的干扰。也有部分报道利用高分辨率的四极杆飞行时间质谱(Q-TOF)^[25,26]^检测,但其分辨率不及四极杆/静电场轨道阱高分辨质谱(Q/Orbitrap HRMS)。Q-Exactive^TM^组合型四极杆/静电场轨道阱质谱仪将四极杆的母离子高性能选择性与静电场轨道阱高分辨的精确质量数相结合,能在单次分析中定性、定量检测复杂混合物中痕量水平的代谢物、污染物、肽类和蛋白质。与三重四极杆质谱多反应监测(MRM)模式不同,Q-Exactive^TM^质谱仪在全扫描/数据依赖二级扫描(Full mass-ddMS^2^)模式下进行一级质谱全扫描的同时,对一级质谱中丰度高的母离子进行二级质谱扫描,可快速定性。在目标离子选择性扫描(Targeted-SIM)模式下,利用目标物母离子的精确质荷比直接定量,无需对目标物逐个优化子离子及相关参数,降低检测时间的同时又能很好地避免受基质干扰而产生假阳性现象。在二级质谱模式下,可直接比对碎片离子精确质荷比,在无标准品的情况下其定性结果也具有一定的可信度,在突发食物中毒事件时,更加有助于样品的快速筛查和定量分析^[27⇓-29]^。目前鲜有运用四极杆/静电场轨道阱高分辨质谱技术对鹅膏肽类毒素进行鉴定检测的研究,近两年开始有文献报道但所涉及的鹅膏肽类毒素种类较少^[30,31]^。本研究建立的毒蘑菇中5种鹅膏肽类毒素定性定量检测的方法,前处理过程简便,分析时间短,测定结果准确,为毒蘑菇中毒的快速确证分析提供了参考依据。

## 1 实验部分

### 1.1 仪器、试剂与材料

UltiMate 3000高效液相色谱仪(美国Dionex公司); Q-Exactive^TM^四极杆-静电场轨道阱高分辨质谱仪、Multifuge X1R离心机(美国Thermo公司); Milli-Q Integral 3超纯水系统(美国Millipore公司)。

*α*-鹅膏毒肽(纯度≥90%)、*β*-鹅膏毒肽(纯度≥90%)、*γ*-鹅膏毒肽(纯度≥90%)、二羟鬼笔毒肽(纯度≥90%)、羧基二羟鬼笔毒肽(纯度≥90%)均购自美国Enzo公司;乙腈和甲醇均为色谱级(纯度≥99.9%),乙酸铵为质谱级(纯度≥99.9%),均购自美国Thermo公司;甲酸为色谱级(纯度≥98%),购自上海安谱实验科技股份有限公司;氨水(质量分数25%),购自德国Merck公司。

### 1.2 标准溶液配制

分别称取5种鹅膏肽类毒素标准品于10 mL容量瓶中,用甲醇溶解并定容至刻度,配制成质量浓度为1000 mg/L的标准储备溶液,于-20 ℃避光保存。分析时以乙腈-水(50∶50, v/v)溶液逐级稀释,配制1.0~20.0 μg/L的系列混合标准溶液。

### 1.3 样品制备

新鲜野生菌样品经组织匀浆机匀浆后,于-20 ℃保存,准确称取匀浆后的试样1 g(精确至0.01 g)于15 mL聚丙烯刻度离心管中,加入20 mL超纯水,涡旋混匀30 s,超声提取10 min, 12000 r/min离心5 min,取上清液过0.22 μm聚四氟乙烯(PTFE)滤膜后,上机分析。

### 1.4 色谱条件

ACQUITY UPLC HSS T3色谱柱(100 mm×2.0 mm, 2.1 μm);流速:0.3 mL/min;柱温:40 ℃;流动相:A为5 mmol/L乙酸铵水溶液(含0.1%甲酸), B为乙腈。梯度洗脱:0~1 min, 5%B; 1~9 min, 5%B~55%B。进样体积:5.0 μL。

### 1.5 质谱条件

离子源:可加热电喷雾离子源(HESI);喷雾电压:3.50 kV;毛细管温度:350 ℃;加热温度:350 ℃;鞘气:45 arb;辅助气:11 arb;扫描模式1: Full mass-ddMS^2^,正离子采集模式,Full mass分辨率70000 FWHM, ddMS^2^分辨率17500 FWHM;扫描模式2: Targeted-SIM,正离子采集模式,分辨率70000 FWHM。各个化合物的质谱信息见表1。

**表1:** 5种鹅膏肽类毒素的质谱信息

Compound	[M+H]^+^	Retention time/min	Theoretical exact mass (m/z)	Actual exact mass (m/z)	Normalized collision energy/eV	Fragment ions (m/z)	Mass difference/ 10^-6^
α-Amanitin	C_39_H_54_N_10_O_14_S	5.39	919.36144	919.36096	25	259.12884, 86.06051	0.52
β-Amanitin	C_39_H_53_N_9_O_15_S	5.29	920.34546	920.34565	25	259.12856, 86.06047	0.21
γ-Amanitin	C_39_H_54_N_10_O_13_S	5.90	903.36653	903.36584	25	243.13386, 86.06046	0.76
Phalloidin	C_35_H_48_N_8_O_11_S	6.57	789.32360	789.32394	28	330.14451, 185.09318	0.43
Phallacidin	C_37_H_50_N_8_O_13_S	6.61	847.32908	847.32863	28	330.14438, 185.09237	0.53

## 2 结果与讨论

### 2.1 色谱条件的选择

鹅膏毒肽和鬼笔毒肽为多肽类环状化合物,具有较强极性,本方法采用能够增强对极性分子反相保留的HSS T3高强度硅胶基质色谱柱分离,考察了甲醇-水体系和乙腈-水体系在梯度洗脱条件下对5种鹅膏肽类毒素峰形、分离度和灵敏度的影响。水体系分别采用0.05%氨水水溶液、0.1%甲酸水溶液、5 mmol/L乙酸铵水溶液(含0.1%甲酸)。结果表明,甲醇-水体系中部分化合物的色谱峰存在不同程度的拖尾、展宽现象,乙腈-水体系可显著改善化合物的峰形,不同化合物的灵敏度均优于甲醇-水体系。在乙腈-水体系中,当水体系为0.05%氨水水溶液时,*α*-鹅膏毒肽*m/z*919.36096的分子离子峰的响应大幅降低,同一保留时间出现丰度较高的*m/z* 917.34768离子峰,根据精确质荷比计算得到其为[C_39_H_53_N_10_O_14_S]^+^^[32]^,推测为*α*-鹅膏毒肽脱去2个氢的产物,由于其丰度较高,[M+H]^+^分子离子峰的形成受到抑制,与之结构相似的*γ*-鹅膏毒肽也有类似现象。对于二羟鬼笔毒肽和羧基二羟鬼笔毒肽,氨水体系中除*m/z* 789.32394和*m/z* 847.32863的峰外,还出现了与[M+H]^+^分子离子峰丰度相当的[M+Na]^+^峰(*m/z* 811.30837和*m/z* 869.31504),推测碱性条件下,化合物中的O、S原子容易捕获Na^+^而形成带电粒子,形成[M+Na]^+^的概率增加,使[M+H]^+^分子离子峰的形成受到抑制,一定程度下影响了检测的灵敏度。当水体系为0.1%甲酸水溶液、5 mmol/L乙酸铵水溶液(含0.1%甲酸)时,由于H^+^浓度更高,二羟鬼笔毒肽和羧基二羟鬼笔毒肽的[M+Na]^+^峰受到极大抑制,丰度降至[M+H]^+^峰的10%以下。但乙酸铵-甲酸体系的分离度略优于甲酸体系,故选用乙腈-5 mmol/L乙酸铵水溶液(含0.1%甲酸)进行分离,图1为5种鹅膏肽类毒素混合标准溶液的提取离子色谱图。

**图1:**
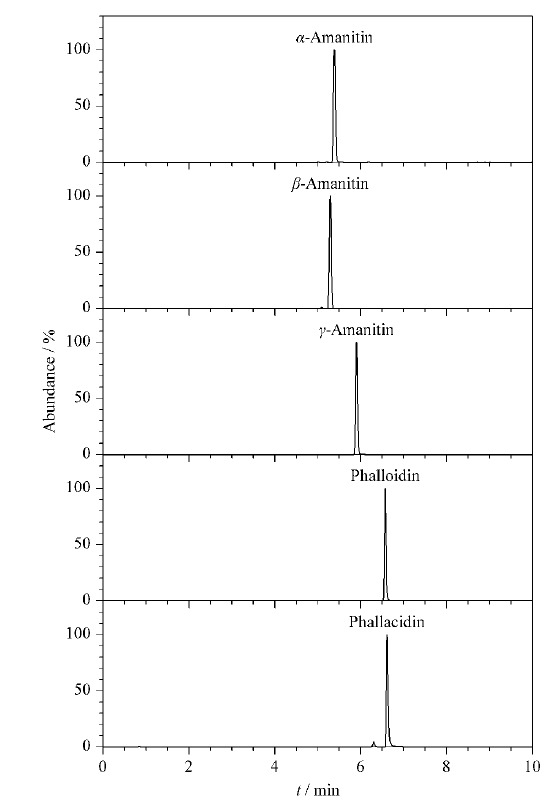
5种鹅膏肽类毒素混合标准溶液(100 μg/L)的提取离子色谱图

### 2.2 质谱条件的选择

*α*-鹅膏毒肽和*β*-鹅膏毒肽的分子质量相差1 Da,且具有相同的子离子,根据^13^C在自然界中的天然丰度计算,含有^13^C的*α*-鹅膏毒肽同位素峰的丰度约为不含^13^C的43%,当*α*-鹅膏毒肽分子含有一个^13^C时(分子式为^13^C_1_C_38_H_54_N_10_O_14_S),其[M+H]^+^峰*m/z*为920.35936,而*β*-鹅膏毒肽的[M+H]^+^峰*m/z*也为920.34565,因此*α*-鹅膏毒肽的同位素峰会对*β*-鹅膏毒肽的检测造成干扰,在低分辨质谱上无法区分,必须尽量通过液相色谱实现基线分离。在使用高分辨质谱时,*α*-鹅膏毒肽和*β*-鹅膏毒肽精确质荷比的差异使得两者在提取离子色谱图和一级质谱图中出峰互不干扰,故对色谱分离条件更为宽容。

为使各化合物更好地离子化,采用流动注射进样方式,将500 μg/L的各化合物标准溶液直接注入质谱仪。在Full mass模式下,根据目标化合物分子式组成,推算其分子离子峰的理论质荷比,与实测值比较,一般两者的质量误差应小于5×10^-6^。同时在Full mass-ddMS^2^模式下,通过色谱分离,优化各化合物的归一化碰撞能(NCE),得到化合物的二级质谱碎片,选择丰度较高的2个碎片离子作为定性特征离子。

5种鹅膏肽类毒素的分子式、保留时间、理论和实测精确质量数、主要碎片离子及质量误差等质谱信息见表1,其一级质谱及二级碎片离子质谱图见图2。

**图2:**
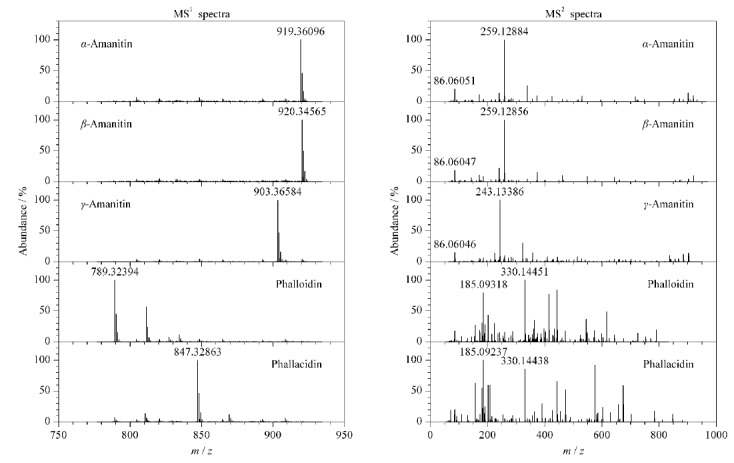
5种鹅膏肽类毒素的一级质谱及二级碎片离子质谱图

### 2.3 质谱碎裂途径

鹅膏毒肽和鬼笔毒肽的分子结构是由氨基酸通过酰胺键首尾连接形成的环肽毒素,相对S-C键,氨基酸之间的肽键更不稳定,因此,高碰撞能量下的裂解一般发生在氨基酸之间的酰胺键上^[32]^。根据Mass Frontier 7.0结构鉴定软件解析5种鹅膏肽类毒素的二级质谱,结合碎片离子精确质荷比,推测其碎裂途径见图3,结构式浅色部分为可能丢失的断裂结构,下方为该化合物二级碎片离子的质量数。*α*-鹅膏毒肽与*β*-鹅膏毒肽具有相同的骨架结构,仅在R_2_取代基上有氨基与羟基的不同,*α*-鹅膏毒肽得到相对丰度较强的*m/z* 259.12284、*m/z* 86.06051质谱峰,*β*-鹅膏毒肽得到相对丰度较强的*m/z* 259.12856、*m/z* 86.06047质谱峰。*γ*-鹅膏毒肽除*m/z* 86.06046质谱峰外,有相对丰度较强的*m/z* 243.13386质谱峰,其与*α*-鹅膏毒肽、*β*-鹅膏毒肽的*m/z* 259.12284、*m/z* 259.12856质谱峰相差16 Da, *γ*-鹅膏毒肽R_1_为一个氢,与*α*-鹅膏毒肽分子式相差一个氧原子,这也佐证了碎裂途径2。二羟鬼笔毒肽和羧基二羟鬼笔毒肽均可得到相对丰度较强的*m/z* 185.09318、*m/z* 330.14451质谱峰,羧基二羟鬼笔毒肽可得到相对丰度较强的*m/z* 185.09237、*m/z* 330.14438质谱峰。

**图3:**
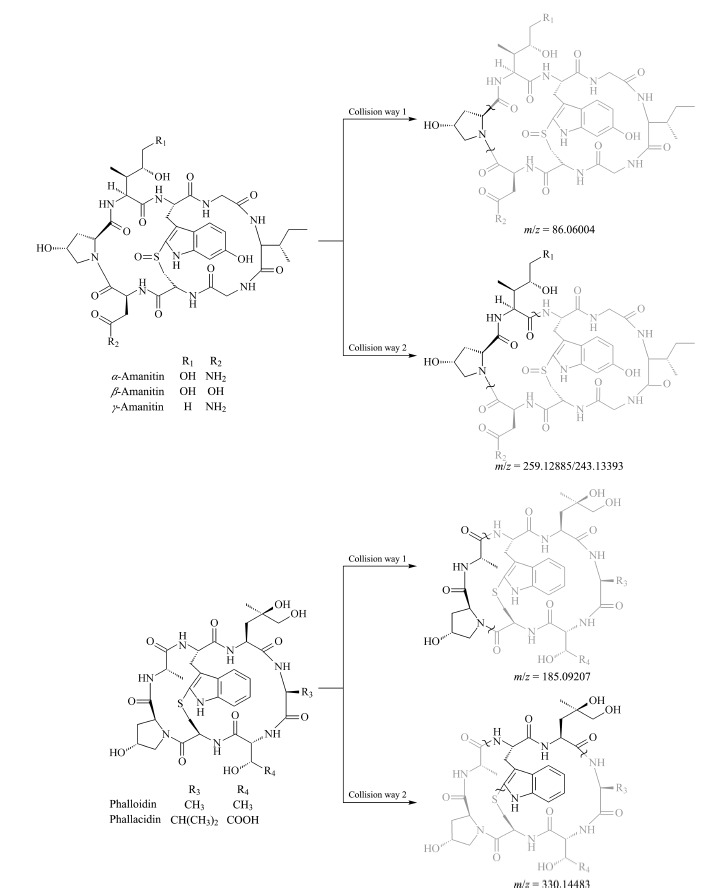
5种鹅膏肽类毒素的结构式及其二级质谱碎裂途径

### 2.4 前处理条件的选择

鹅膏毒肽和鬼笔毒肽的分子结构中含有较多羟基、氨基等,极性较强,易溶于水、甲醇、乙腈等溶剂,发生急性中毒时需要以最快的速度进行定性和定量检测,所以不宜采用固相萃取等前处理时间较长的净化方法。故尝试以纯水、甲醇、甲醇-水(50∶50, v/v)、乙腈、乙腈-水(50∶50, v/v)作为提取溶剂,在预先加标的空白样品中对目标物进行提取,分析不同提取条件下5种鹅膏肽类毒素的响应值和回收率。结果显示,纯乙腈提取进样时,样品提取溶剂与流动相不一致而产生了溶剂效应,导致*α*-鹅膏毒肽与*β*-鹅膏毒肽色谱峰有前伸现象。其余提取试剂的响应较好,回收率均大于80%,且未有明显的基质效应,符合中毒事件快速定性要求。考虑到对环境影响较小,故选择纯水作为提取试剂。当离心速度为5000 r/min时,水提取液有混浊现象,提高到12000 r/min后可得到澄清溶液,故将离心速度设定为12000 r/min。

### 2.5 基质效应的研究

基质效应(matrix effect, ME)是指从样品中与目标物同时提取出来的物质引起的目标物分析信号抑制或增强的现象,ME值可按下式进行量化评估^[33]^。

ME= $\left(\frac{基质溶液中目标物峰面积}{纯溶剂目标峰面积}-1\right)$ ×100%

ME为正值时,基质效应表现为增强效应,反之减弱。当ME值小于20%时,则认为基质效应对定量分析没有显著影响,可以忽略。*α*-鹅膏毒肽、*β*-鹅膏毒肽、*γ*-鹅膏毒肽、二羟鬼笔毒肽和羧基二羟鬼笔毒肽的ME分别为-10.8%、-11.9%、-11.3%、-9.5%、-10.6%,可见5种鹅膏肽类毒素的基质抑制效应可以忽略。

### 2.6 方法的线性关系、检出限与定量限

精确配制1、2、5、10、15、20 μg/L的5种化合物混合标准工作液,Targeted-SIM模式下测定。以化合物的质量浓度(*X*, μg/L)为横坐标,化合物准分子离子色谱峰面积(*Y*)为纵坐标制作标准曲线,得出线性范围、回归方程和相关系数(*r*),结果见表2。5种鹅膏肽类毒素在1.0 ~20.0 μg/L范围内线性关系良好,相关系数为0.9974~0.9989。以化合物准分子离子色谱峰的3倍和10倍信噪比(*S/N*)对应样品中目标物的浓度,作为方法的检出限(LOD)和定量限(LOQ)。结果表明,5种鹅膏肽类毒素的检出限为0.006 mg/kg,定量限为0.02 mg/kg。

**表2:** 5种鹅膏肽类毒素的线性范围、回归方程、相关系数、检出限及定量限

Compound	Regression equation	Linear range/(μg/L)	r	LOD/(mg/kg)	LOQ/(mg/kg)
α-Amanitin	Y=1.75×10^4^X+1.74×10^3^	1.0-20.0	0.9985	0.006	0.02
β-Amanitin	Y=1.03×10^4^X+1.67×10^3^	1.0-20.0	0.9974	0.006	0.02
γ-Amanitin	Y=2.69×10^4^X+1.61×10^3^	1.0-20.0	0.9982	0.006	0.02
Phalloidin	Y=2.94×10^4^X+3.68×10^3^	1.0-20.0	0.9986	0.006	0.02
Phallacidin	Y=3.57×10^4^X+7.04×10^3^	1.0-20.0	0.9989	0.006	0.02

Y: peak area; X: mass concentration, μg/L.

### 2.7 回收率与精密度

取空白蘑菇样品进行加标回收试验,分别添加低、中、高3个水平的混合标准物质,每个水平重复测定6次,考察方法的准确度(回收率)和精密度,结果如表3所示。5种鹅膏肽类毒素的回收率为81.8%~102.4%,其相对标准偏差(RSD)为3.2%~8.3%。

**表3:** 5种鹅膏肽类毒素的平均加标回收率及相对标准偏差(n=6)

Compound	1.0 μg/L		5.0 μg/L		20.0 μg/L	
	Recovery/%	RSD/%	Recovery/%	RSD/%	Recovery/%	RSD/%
α-Amanitin	84.5	7.1	87.5	6.8	92.0	6.4
β-Amanitin	84.8	5.8	90.1	5.2	93.0	3.4
γ-Amanitin	81.8	8.3	90.7	7.9	93.6	6.5
Phalloidin	89.1	7.0	90.7	3.2	92.4	3.9
Phallacidin	83.6	7.4	89.2	7.8	102.4	6.2

### 2.8 样品检测

#### 2.8.1 样品的定性定量分析

应用本方法对采集的肉褐鳞环柄菇进行检测,样品按1.3节制备后进样分析。5种鹅膏肽类毒素的提取离子色谱图见图4,该样品有与*α*-鹅膏毒肽、*β*-鹅膏毒肽保留时间一致的色谱峰,*γ*-鹅膏毒肽、二羟鬼笔毒肽和羧基二羟鬼笔毒肽的提取离子未见相应的色谱峰。图4也提供了样品中*α*-鹅膏毒肽、*β*-鹅膏毒肽的一级质谱和二级碎片离子质谱图,样品和标准溶液相比,*α*-鹅膏毒肽、*β*-鹅膏毒肽一级质谱和二级碎片离子的质量误差均小于5×10^-6^。样品中检出的*α*-鹅膏毒肽、*β*-鹅膏毒肽含量分别为347.1 mg/kg和92.7 mg/kg。

**图4:**
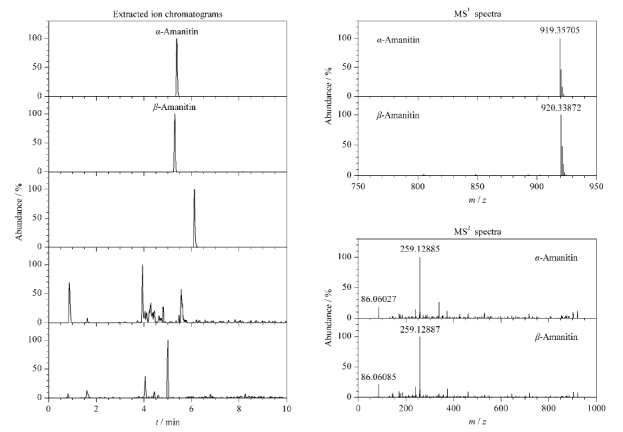
样品中鹅膏肽类毒素的提取离子色谱图及*α*-鹅膏毒肽、*β*-鹅膏毒肽的一级质谱和二级碎片离子质谱图

#### 2.8.2 样品中未知物的结构分析

图4中有一化合物保留时间为6.13 min,该化合物一级质谱及二级碎片离子质谱图见图5。由于无标准品进行比对,根据其[M+H]^+^准分子离子峰(*m/z* 903.36648)推测其为*γ*-鹅膏毒肽的同分异构体。

**图5:**
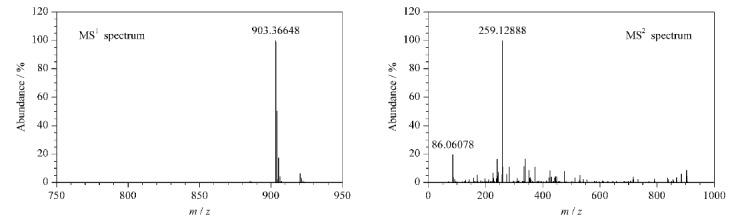
未知化合物的一级质谱和二级碎片离子质谱图

该化合物的分子质量为902.3 Da,分子结构上比*α*-鹅膏毒肽少一个羟基。该化合物含有*m/z* 259.12888和*m/z* 86.06078碎片离子质谱峰,与*α*-鹅膏毒肽和*β*-鹅膏毒肽碎片离子一致,推测其结构与碎裂规律两者类似。结合文献^[34]^报道的三羟基鹅膏毒肽酰胺(amaninamide)结构及碎裂规律推断,该未知化合物可能的结构及质谱碎裂途径见图6。

**图6:**
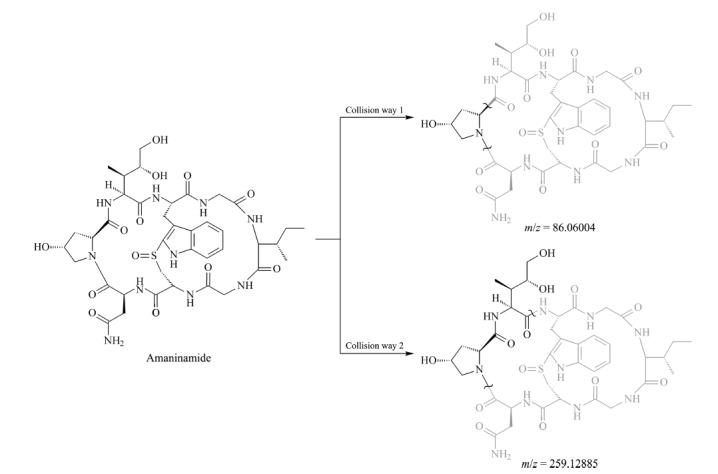
样品中未知化合物推测的结构式及碎裂途径

由此可见,利用高分辨质谱的精确质荷比以及二级质谱碎片信息,在没有标准品的情况下,可进行未知化合物的结构推断,为快速锁定化合物提供依据。

## 3 结论

本研究利用超高效液相色谱-四极杆/静电场轨道阱高分辨质谱建立了毒蘑菇中5种鹅膏肽类毒素的检测方法。方法准确、快速,灵敏度高,可同时进行定性定量测定,并可延伸至未知化合物的结构推断。在此基础上,可进一步建立鹅膏肽类毒素非靶向筛查方法和筛查数据库,为突发公共卫生事件应急检测提供可靠的技术支持。
